# Electrophysiological Correlates of Response Time Variability During a Sustained Attention Task

**DOI:** 10.3389/fnhum.2019.00363

**Published:** 2019-10-15

**Authors:** Keitaro Machida, Michael Murias, Katherine A. Johnson

**Affiliations:** ^1^Melbourne School of Psychological Sciences, The University of Melbourne, Melbourne, VIC, Australia; ^2^Institute for Innovations in Developmental Sciences, Northwestern University, Chicago, IL, United States

**Keywords:** sustained attention, EEG, ADHD (attention deficit and hyperactivity disorder), response time variability, graph theory

## Abstract

Individuals with Attention Deficit Hyperactivity Disorder (ADHD) tend to perform cognitive tasks with greater Response Time Variability (RTV). Greater RTV in ADHD may be due to inefficient functional connectivity of the brain during information processing. This study aimed to investigate the relationship between brain connectivity, RTV, and levels of ADHD symptoms. Twenty-eight children aged 9–12 years and 49 adolescents aged 15–18 years performed the Sustained Attention to Response Task (SART) while EEG was recorded. The participants’ levels of ADHD symptoms were measured using self- and parent-rated questionnaires. The ex-Gaussian analysis and The Fast Fourier Transform were used to measure multiple aspects of RTV. Functional connectivity between 64 electrodes was computed during task performance, and global efficiency and modularity were calculated, reflecting integration and segregation of the brain, respectively. There was a positive association between multiple RTV measures and the level of ADHD symptoms, where participants with higher levels of ADHD symptoms showed greater RTV, except for sigma from the ex-Gaussian analysis. More efficient brain network activity, measured by global efficiency, was associated with reduced RTV. Children showed greater RTV and less efficient brain network activity compared with the adolescents. These findings support the view that stable responses are achieved with more integrated (and efficient) brain connectivity.

## Introduction

ADHD is a neurodevelopmental disorder characterized by very high levels of inattention, hyperactivity, and impulsivity (American Psychiatric Association, 2013). Higher variability in responding to targets during cognitive tasks is a ubiquitous finding in ADHD studies (Leung and Connolly, 1996; Oosterlaan and Sergeant, 1996; Scheres et al., 2001; Stevens et al., 2002). Researchers have attempted to determine the underlying cognitive mechanisms associated with this increased Response Time Variability (RTV), most often using methods focused on patterns within the response time data itself (Karalunas et al., 2014). The standard deviation of response time (SDRT) is the most common among a series of statistical models used to examine RTV patterns and their links with cognition. While these RTV models are extremely useful, they do not clarify the brain mechanisms underlying this increased RTV. An evaluation of brain activity patterns associated with increased RTV will help elucidate the biological origins of RTV in people with high symptoms of ADHD.

One widely-used RTV model is the ex-Gaussian distribution model, which provides an excellent fit to RT data (Luce, 1986). It expresses the RT distribution as the sum of both the normal and exponential distributions. The advantage of using the ex-Gaussian distribution model is that RT distributions are often positively skewed and the exponential portion models this skew very well (Luce, 1986). Three parameters are used to describe the RT distribution: mu, sigma, and tau. Mu is calculated as the mean, and sigma is calculated as the standard deviation of the Gaussian distribution, while tau defines the mean of the exponential distribution. Greater RTV, expressed as sigma and tau in the model, has often been observed in ADHD populations (Leth-Steensen et al., 2000; Hervey et al., 2006; Buzy et al., 2009; Vaurio et al., 2009). Often the ADHD group’s RT distribution is largely skewed compared with the control group indicating that there are larger numbers of abnormally slow responses (Castellanos et al., 2006). Leth-Steensen et al. (2000) suggested that greater tau indicates a greater number of lapses in attention. The authors implied that a greater number of lapses in attention might explain why greater RTV is very common in people with ADHD.

Another approach used to analyze RTV is the fast Fourier transform (FFT), allowing for the capture of periodic fluctuations in RT. The FFT measures the power of periodic changes in RT at different temporal frequencies. This analysis of periodic fluctuation has been applied to RT data from individuals diagnosed with ADHD (Castellanos et al., 2005) as they have performed the Change Task (Geurts et al., 2008), simple and complex Go/No-go Tasks (Vaurio et al., 2009), and the Stopping Task (Karalunas et al., 2013). These studies used the frequency bands identified by Buzsaki and Draguhn (2004) that are based on neuronal rhythms. The majority of studies found that people with ADHD performed the tasks with significantly greater RTV in the “slow 4” (0.027–0.074 Hz) frequency band. This frequency band is closely related to resting state brain activity (Greicius et al., 2003; Sonuga-Barke and Castellanos, 2007). Greater RTV in this frequency band might imply that people with ADHD are failing to suppress this resting state activity during tasks, leading to more variable responses (Sonuga-Barke and Castellanos, 2007). There are still inconsistencies, however, around which frequency bands have been used to measure RTV and the interpretation of what each band reflects (Karalunas et al., 2013).

An alternative way to define frequency bands of RTV is by dividing frequency based on the experimental paradigm. This was the approach taken by Johnson et al. (2007) with data from the Sustained Attention to Response Task (SART) originally developed by Robertson et al. (1997). In the fixed version of the SART, the digits 1–9 are presented in sequence, 25 times, and participants are asked to respond to all digits except 3. Participants tend to slow their response to digit 1 in anticipation of the upcoming No-Go digit 3. This slowing in response creates a pattern in the RT sequence, resulting in a peak in the FFT spectrum at 0.0772 Hz. This peak was used to divide the RT into a slow and a fast band. The power in the slow frequency band provides a measure of gradual changes in RTs over the course of the task and is argued to reflect the participant’s arousal level. The power in the fast frequency band provides a measure of trial-to-trial variability and is argued to reflect the participant’s moment-to-moment sustained attention (Johnson et al., 2007). The experimental set-up of the fixed SART provides a meaningful way to define the frequency bands for the FFT analysis of RTs. Previous research has shown that individuals with ADHD perform the SART with significantly greater RTV in both the slow and fast bands, indicating deficits in maintaining arousal and sustained attention respectively (Johnson et al., 2007; Adamo et al., 2014).

The neural correlates of RTV have not been widely examined. One explanation of RTV is that increased RTV may reflect inefficient or inconsistent information flow through the brain during performance of a cognitive task (Russell et al., 2006). Stable responses might require information to flow more efficiently and consistently for every trial – variable responses might arise from inefficient or disruptive information processing in the brain. The Cognitive Neuroenergetic model suggests that greater RTV associated with ADHD might be caused by inefficient information processing (Russell et al., 2006). The efficiency of information processing can be measured using the connectivity patterns of the brain.

One method with which to characterize patterns of brain connectivity is to apply graph theoretical analysis. In graph theoretical analysis, the brain is considered as a single network, and this analysis describes the patterns of connections between brain areas (Bullmore and Sporns, 2009; Stam and van Straaten, 2012). The characteristics of the brain’s connectivity and information flow are described in two ways – functional integration and segregation (Bullmore and Sporns, 2009; Sporns, 2013). Functional integration measures the engagement of the global network, where higher integration implies that the whole system is working closely to process information. Functional segregation measures locally segregated processes. Higher segregation implies the presence of a number of different communities within a whole network, with a greater level of information that is processed locally. Previous studies have shown that network reconfiguration of the brain occurs across the lifespan (Cao et al., 2014; Zuo et al., 2017), and within the time scale of milliseconds (Valencia et al., 2008; Cruzat et al., 2016). Studies have shown that adult participants perform cognitive tasks with increased integration and decreased segregation of brain networks when compared with a baseline period, indicating that brain networks reconfigure their connections as appropriate for particular cognitive processes (Bola and Sabel, 2015; Cruzat et al., 2016). We argue that RTV could be explained by how the connections between different areas of the brain are rearranged from the resting to the task active state. Changes in network measures, obtained from graph theoretical analysis during a cognitive task, might reveal whether there are particular connectivity patterns and dynamic configurations that are associated with RTV in people with high and low symptoms of ADHD.

Abnormal brain connectivity in ADHD has been demonstrated using both functional and structural connectivity analyses (Castellanos et al., 2008; Cubillo et al., 2010; Fair et al., 2010; Konrad et al., 2010; Konrad and Eickhoff, 2010; Sidlauskaite et al., 2016). Using graph theoretical analysis with both EEG (Ahmadlou et al., 2012; Liu et al., 2015) and fMRI (Wang et al., 2009; Cao et al., 2014; Beare et al., 2017), it has been shown that individuals with ADHD show decreased functional integration and increased strength of segregation of the brain, as indicated by a disruption in long-range connections, lower global efficiency, and greater modularity. Wang et al. (2015) has also reported that individuals with ADHD exhibited increased modularity and decreased global efficiency using resting state fMRI. These finding suggest that individuals with ADHD might have altered brain networks compared with matched controls. Liu et al. (2015) suggested that adequate levels of integration and segregation of the brain is not achieved for individuals with higher levels of ADHD symptoms. Cao et al. (2014) have also proposed that individuals with ADHD might have a disrupted balance of integration and segregation of the brain, and this might explain the behavioral symptoms of ADHD. In contrast, some other studies using fMRI found no difference in the level of integration measured by global efficiency between individuals with and without ADHD during resting state (Cocchi et al., 2012; Sato et al., 2013). As the majority of studies are based on resting state data rather than task performance it is critical to examine brain activity during a task and how levels of integration and segregation relate to ADHD symptoms and behavioral performance. It might be the case that decreased functional integration and strengthened segregation of the brain may explain increased RTV in ADHD.

When examining the relationship between RTV and ADHD symptoms it is critical to examine how the relationship changes from childhood to adolescence. During typical development RTV decreases, on average, from childhood to young adulthood, and young adulthood is associated with the most stable responses during the lifespan (Williams et al., 2005; Dykiert et al., 2012). With this decreasing trend of RTV, the association between RTV and ADHD symptoms might also alter as children enter into adolescence. McAuley et al. (2014) showed that the difference in RTV between their ADHD and control groups was only present in children and not in adolescents. This implies that increased RTV in ADHD might be a phenomenon specific to children with ADHD. According to the meta-analytic review by Kofler et al. (2013), however, both children and adults with ADHD showed increased RTV compared to age-matched groups, but the effect size was smaller in adults compared with children. This suggests that the relationship between ADHD symptoms and RTV may be changing from childhood to adolescence, where the relationship might be weakening over time.

The symptoms of ADHD are distributed continuously within the population from extremely low to high levels of inattention, hyperactivity, and impulsivity. Individuals with ADHD populate the extreme high end of its continuum (Kuntsi et al., 2001, 2009). Here we use the approach of measuring the level of ADHD symptoms shown by each participant, rather than dichotomizing participants into ADHD and control groups. This approach of measuring ADHD symptom levels along the continuum has been used previously in behavioral (Kuntsi et al., 2001; Kollins et al., 2005; Diamantopoulou et al., 2007; Bidwell et al., 2014) and EEG (Broyd et al., 2011) studies. Categorizing an ADHD and a control group by choosing a cut-point at the extreme end of the ADHD symptom distribution will result in variation in levels of ADHD symptoms between individuals within the same group. This group comparison may hinder our understanding of how different levels of ADHD symptoms impact behavior. Frazier et al. (2007), therefore, suggested that more studies should examine ADHD symptoms as a continuous variable in order to further understand the impairments in cognitive processes underlying ADHD.

Individuals with ADHD often, but not always, show difficulty in sustaining their attention. Sustained attention is the conscious processing of non-arousing stimuli over a period of time (Robertson et al., 1997). Behavioral measures that are often used in sustained attention tasks include the number of omission and commission errors made and the speed and consistency of response (Huang-Pollock et al., 2012). A meta-analysis by Huang-Pollock et al. (2012) reported more omission and commission errors and greater RTV measured by SDRT were found in performance of the Continuous Performance Test (CPT) by participants with ADHD relative to those without ADHD, across the ages of 6–12. Using the SART, greater numbers of omission and commission errors, and greater SDRT were also observed in children with ADHD compared with those without ADHD (O’Connell et al., 2004). A meta-analysis by Wright et al. (2014) including both CPT and SART, also reported more errors of omission and commission made by children with ADHD. These findings support the proposition that sustained attention is impaired in individuals with ADHD.

This study aims to investigate the relationship between brain connectivity, RTV, and levels of ADHD symptoms in children and adolescents on two measures of sustained attention. It is hypothesized that (1) Higher levels of ADHD symptoms will be associated with increased RTV; (2) Children will perform the sustained attention tasks with greater RTV than adolescents; (3) Greater functional integration and decreased strength of segregation of the brain will be associated with reduced RTV and lower levels of ADHD symptoms; and (4) Adolescents will show more integrated and more strongly segregated patterns of brain connectivity than children.

## Materials and Methods

### Participants

Twenty-eight children aged 9–12 years (*M* = 10.95 years, *SD* = 1.06; 9 females) and 49 adolescents aged 15–18 years (*M* = 17.57 years, *SD* = 0.76; 33 females) participated in this study. One participant from the adolescent group was removed from the sample before analysing the data due to excessive movement during the EEG recording, resulting in a total of 49 adolescents from the initial number of 50 adolescents. Children were recruited from local primary schools. Six adolescents were recruited from local secondary schools and forty-three were recruited from the university’s first year Psychology course. Participants were not pre-selected based on symptoms of ADHD.

### Materials

#### The Sustained to Attention to Response Task (SART)

The experimental paradigm was created using Matlab (Mathworks Inc.) and Psychtoolbox (Kleiner et al., 2007). The experimental paradigm followed the same procedure as a study by Johnson et al. (2007). Both the Fixed and Random versions of the Sustained Attention to Response Task (SART) were used. Participants were presented a series of digits (1–9) on a computer screen. Each trial was set-up with this timing: a single digit was presented for 313 ms, followed by a mask for 125 ms. A response cue was presented for 63 ms, and then a second mask was presented for 375 ms. Finally, a fixation cross was presented for 563 ms. The total inter-stimulus interval was 1439 ms. Participants were asked to respond to every digit except “3.” In the Fixed version of the SART, the digits were presented in the order from “1” to “9,” and this cycle was repeated 25 times. In the Random version the order of the digit was randomized, although the no-go digit “3” was never presented twice in a row. The participants were asked to respond when the response cue appeared on screen to limit impulsive response styles shown by children and some people with high ADHD symptoms. Each SART consisted of 225 trials, lasting approximately 5.5 min. The order of presentation of the two SARTs was counterbalanced across the participants.

#### Conners 3 ADHD Index

The Conners 3 questionnaire was used to measure levels of ADHD symptoms. Two forms were used in this study: The Conners 3 parent form was completed by parents of all children and the 6 adolescents recruited through secondary schools, while the self-report form was completed by the university-based adolescents. There was no significant difference between the adolescent (M = 51.00, SD = 12.90, range: 41–90) and child (M = 56.61, SD = 15.08, range: 43–90) groups on the ADHD Index, t(49.11) = –1.65, p = 0.10. The correlation between the parent and self-report forms is 0.57 for the ADHD Index (Conners, 2008). Eight children (29%) and nine adolescents (18%) had ADHD Index scores at 65 and above, which is indicative of elevated levels of ADHD symptoms.

#### Wechsler Abbreviated Scale of Intelligence (WASI)

An estimate of intelligence quotient (IQ) was obtained using the WASI (Wechsler, 2011) for participants recruited from the primary and secondary schools to ensure they were capable of understanding the task instructions. Participants were to be excluded if their IQ was below 70: no participants were excluded. Participants recruited from the university were assumed to have a FSIQ above 70.

### Procedure

Participants were asked to complete the SARTs while the EEG was recorded using a 64 electrode Biosemi system. For participants recruited from schools, the WASI was completed before the SART. Parents were asked to complete the Conners 3 questionnaires while the participants were performing the task. The undergraduate participants were asked to complete the Conners 3 questionnaires form while the EEG was being set up.

### Analysis

#### Behavioral Analysis

For each participant, for the Fixed and Random SARTs separately, each dependent variable was computed following these steps. A count of the two types of errors was computed. Omission errors are the missed responses to the Go trials. Commission errors are the responses to the No-Go trials. Participants with more than 30 omission errors in either Fixed or Random SART were to be removed from the analysis: no participants were excluded. Trials containing commission errors, response times shorter than 100 ms, and No-Go trials were removed, and subsequently the Mean of RT and Standard Deviation of RT (SDRT) were computed. Three parameters, mu, sigma, tau were extracted by applying the ex-Gaussian model based on the approach by Lacouture and Cousineau (2008).

The analysis to compute fast and slow RTV followed the procedure of Johnson et al. (2007) with some modifications (see below). Removed trials were linearly interpolated. The RT data were analyzed according to Welch’s averaged, modified periodogram method. Trials were divided into 7 segments of 75 trials of the SART, with a 50 trial overlap. Each segment was detrended, Hamming-windowed, and zero padded to 450 data points. The FFT was then applied to each segment, and the segments were averaged to provide a spectrum per participant. Any segment of 75 data points where there were 5 or more consecutive interpolated trials was excluded in the FFT. If more than 3 segments were removed, the participant’s RT data was excluded from the FFT analysis. The Random SART data of one child were excluded for this reason, and were treated as missing values.

The frequency range was divided into fast and slow frequency bands using the peak at 0.0772 Hz. The frequency 0.0772 Hz was chosen as it is the reciprocal of one cycle of the 1–9 digit presentation in the Fixed SART (a SART cycle). The Fast Frequency Area Under the Spectra (FFAUS) encompassed all sources of variability faster than once per SART cycle, the area under the curve to the right of the peak at 0.0722 Hz. The Slow Frequency Area Under the Spectra (SFAUS) encompassed all sources of variability slower than once per SART cycle: the area under the curve to the left of the peak at 0.0772 Hz.

#### EEG Analysis

The EEG was recorded using the 64 electrode BioSemi system with a sampling rate of 512 Hz. In the BioSemi system, a Common Mode Sense (CMS) active electrode placed on the surface of the head was used as a reference electrode during the recording. The recorded EEG with 64 electrodes was imported and pre-processed in Matlab and Fieldtrip (Oostenveld et al., 2011).

In the software, the EEG data were first referenced to an average of the two ear electrodes. The data were band pass filtered from 0.5 to 40 Hz, and then segmented into trials from -200 to 1200 ms, where 0 was set at the presentation of the target digit of the SART. The data were visually inspected and trials with artifacts such as extraordinary large amplitudes, jumps, and flat signals were removed. Noisy electrodes were manually identified and temporally separated from the data. Independent Component Analysis (ICA) was then applied to remove artifacts such as eye blinks, eye movements, and heartbeats. After ICA, noisy electrodes temporally separated in the previous step were interpolated using the spherical spline method and noisy trials, with amplitudes above 100 and below 100 microvolts, were manually removed from further analysis. The data were then referenced to a common average of the electrodes and detrended. Trials removed for the behavioral analysis were also removed in the EEG analysis, which included incorrect responses, No-Go trials, and trials with less than 100 ms responses.

The analysis followed the event related networks approach by Valencia et al. (2008) and Vourkas et al. (2011). The time frequency analysis was applied from 1 to 40 Hz in steps of 1 Hz and from -200 to 1200 ms in steps of 10 ms using the Complex Morlet Wavelet (Cohen, 2014). Phase information was extracted for each step of frequency and time, which were computed by the time frequency analysis. Functional connectivity was computed using Phase Lag Index (PLI) and connectivity matrices were produced (Stam et al., 2007). PLI measures the asymmetry of how phase differences of two signals are distributed, where greater asymmetry reflects stronger functional connection between two signals. The values of PLI range from 0 to 1, where a higher value represents stronger connections. Computed connectivity matrices from PLI for each step of time and frequency were treated as weighted and undirected networks without thresholding. By setting a threshold, only connections stronger than the threshold can be present in the connectivity matrix, which helps to reduce noisy and spurious connections. One potential concern of this approach is that the single arbitrary threshold or a range of thresholds need to be used, but there is no standard threshold and using different thresholds can lead to different results (Stam and van Straaten, 2012). Even though treating the connectivity matrix as a weighted network can pose a several concerns that the analysis involves noisy and weak connections, this approach can avoid setting an arbitrary threshold. Using connectivity matrices treated as weighted networks, global efficiency and modularity were computed for each step of frequency and time using Brain Connectivity Toolbox (Rubinov and Sporns, 2010). Global efficiency is the inverse of the average shortest path length in the network and a measure of functional integration, reflecting how closely each node is connected. Modularity is measuring a strength of functional segregation and reflects how well a network can be divided up into smaller sub-communities. These two measures were averaged to three frequency ranges, theta (4–8 Hz), alpha (8–13 Hz), and beta (13–30 Hz) bands (Yu et al., 2016). In addition, two time periods were selected *a priori* to measure global efficiency and modularity during the baseline and task. The baseline period was from -200 to 0 ms and global efficiency and modularity were averaged over this period to produce baseline values separately for the three frequency bands. These two measures were baseline corrected by subtracting the baseline values, and the maximum absolute change from baseline was identified during the period between 30 and 600 ms after the digit presentation. This peak value reflects how large the two network measures change from the baseline: this could be either positive or negative depending on the direction of change. The period from 300 to 600 ms was selected for the task period. The brain is thought to be involved in higher cognitive processes during this period (Polich, 2007; Ramchurn et al., 2014; Bola and Sabel, 2015). This analysis resulted in measures of global efficiency and modularity at the three frequencies (theta, alpha, beta) during the task period for the Fixed and Random SARTs separately. The statistical results for the baseline period were summarized in a table in Supplementary Materials.

#### Statistical Analysis

Statistical analysis was performed in R, using linear mixed effect models (LMEM) with random intercepts. LMEM allows data to have missing values. The behavioral measures of this study were the number of omission and commission errors, mean RT, SDRT, mu, sigma, tau, FFAUS, and SFAUS. The network measures were global efficiency and modularity in three frequency bands (theta, alpha, and beta) during the task. Additionally, there were three other variables; SART Task (fixed and random), Age (adolescent and child groups) and the ADHD Index scores.

To examine the relationships between behavioral measures, ADHD Index, and network measures, three different types of models were examined. Two categorical variables (Age and SART Task) were inserted into all three types of models as predictors. The first type of model aimed to examine the association between the behavioral measures and the ADHD Index. Each behavioral measure was treated as an outcome variable, and the ADHD Index and the two categorical variables were the predictors. A single model was fitted for each behavioral measure. In addition to the association between the ADHD Index and the behavioral measures, the effects and interactions of the two categorical variables on behavioral performance were also examined. In the second type of model, the association between the behavioral measures and the network measures was examined. Each behavioral measure was treated as an outcome variable, and each single network measure was inserted as a predictor, along with the two categorical variables. A single model was fitted for each behavioral measure and network measure. In addition to the association between the behavioral measures and the network measures, interactions between the network measures and the two categorical variables were also examined. In the third type of model, the association between the network measures and the ADHD Index was examined. Each network measure was treated as an outcome variable, and the ADHD Index was inserted as a predictor, along with the two categorical variables. A single model was fitted for each network measure. In addition to the association between the network measures and the ADHD Index, the effects and interactions of the two categorical variables on network measures were also examined.

The predictor coefficients in the models were tested by 95% bootstrap Confidence Interval (95% CI) with 2000 resamples. If the confidence interval did not contain 0, it was considered to be a significant predictor. Pairwise comparisons were performed for the significant interaction terms in the models using the lsmeans package in R (Lenth, 2016). *P*-values were adjusted using the False Discovery Rate (FDR) method (Benjamini and Hochberg, 1995). As this study has more of an exploratory rather than a confirmatory purpose, increasing power was favored compared to strongly controlling for the family wise error rate.

## Results

### Behavioral Measures – ADHD Index, Age, SART Task

In this first type of model, Generalized linear mixed effect models (GLMEM) were used for omission and commission errors with the Poisson distribution, given they are count data. LMEMs were used for the other behavioral measures. Behavioral measures were treated as outcome variables and three predictors were inserted in the models – ADHD Index, Age, and SART Task. ADHD Index was centered to reduce collinearity. Categorical variables, Age, and SART Task were coded using the sum contrasts, where children and the Random SART were coded as -1 and adolescents and the Fixed SART were coded as 1. Statistical results were summarized and reported in Table 1.

**TABLE 1:** Analysis for behavioral measures.

**Outcome variables**	**Predictors**	**β and CI**	**Significant interactions**	**β and CI**	**Significant pairwise comparison**
					
Mean RT	ADHD	β = −0.067, CI = [−1.35, 1.19]			
	Age	β = −48.8, CI = [−66.9, −31.2]^∗^			
	SART	β = 0.65, CI = [−10.6, 11.9]			
SDRT	ADHD	β = 1.19, CI = [0.556, 1.80]^∗^			
	Age	β = −26.0, CI = [−34.8, −17.2]^∗^			
	SART	β = 2.34, CI = [−1.54, 6.22]			
Mu	ADHD	β = −2.06, CI = [−3.45, −0.650]^∗^			
	Age	β = −35.0, CI = [−55.0, −15.2]^∗^			
	SART	β = −0.110, CI = [−15.4, 13.7]			
Sigma	ADHD	β = −0.275, CI = [−0.828, 0.290]			
	Age	β = −20.1, CI = [−28.3, −11.9]^∗^			
	SART	β = 1.04, CI = [−4.19, 6.43]			
Tau	ADHD	β = 1.99, CI = [1.27, 2.68]^∗^			
	Age	β = −13.7, CI = [−23.6, −3.85]^∗^			
	SART	β = 0.739, CI = [−6.10, 7.39]			
FFAUS	ADHD	β = 3.18, CI = [0.933, 5.25]^∗^			
	Age	β = −70.3, CI = [−102.6, −41.3]^∗^			
	SART	β = 5.01, CI = [−6.79, 16.2]			
SFAUS	ADHD	β = 13.3, CI = [3.59, 23.4]	Age × Flanker (Figure 1)	β = 46.5, CI = [6.23, 87.9]	Child:Con − Child:Inc., *p* = 0.001^∗^
	Age	β = −356.9, CI = [−500.8, −216.9]^∗^			Adol:Con − Adol:Inc., *p* = 0.443
	SART	β = 1.77, CI = [−72.2, 70.4]			
Omission errors	ADHD	β = 0.023, CI = [−0.002, 0.046]			
	Age	β = −0.780, CI = [−1.13, −0.444]^∗^			
	SART	β = 0.055, CI = [0.052, 0.163]			
Commission errors	ADHD	β = 0.006, CI = [−0.004, 0.015]			
	Age	β = −0.539, CI = [−0.676, −0.406]^∗^			
	SART	β = −0.111, CI = [−0.200, −0.024]^∗^			

**ADHD represents the ADHD Index and it is continuous data. For Age, −1 = children or Child, 1 = adolescents or Adol. SART represents the SART Task, and −1 = Random, 1 = Fixed. For Flanker, −1 = Incongruent or Inc., 1 = Congruent or Con. β represents the coefficient of a predictor variable. CI represents 95% Confidence Interval. ^∗^p < 0.05 or CI does not contain 0. GE represents Global Efficiency, Mod represents Modularity, (theta) represents the theta band, (alpha) represents the alpha band, (beta) represents the beta band.**

#### Main Effect of Age on Behavioral Measures

There were significant differences in task performance between the children and adolescents. The adolescent group performed the SARTs with faster mean RT smaller SDRT, faster mu, β = −35.0, 95% CI = [−54.9, −15.2], smaller sigma, smaller tau, smaller FFAUS, smaller SFAUS, and less omission, and commission errors.

#### Main Effect of SART Task on Behavioral Measures

Participants made significantly more commission errors during the Random than the Fixed SART. There were no other significant differences in performance on the fixed and random SARTs.

#### Main Effect of ADHD Index on Behavioral Measures

ADHD Index was positively associated with SDRT, tau, FFAUS, SFAUS, and negatively associated with mu. Other measures were not significantly associated with ADHD Index.

#### Interactions on Behavioral Measures

There were no significant interactions found.

### Behavioral Measures With Network Measures

In this second type of model, LMEMs were used to assess the relationship between the RT and network measures. The behavioral measures of mean RT, SDRT, mu, sigma, tau, FFAUS, and SFAUS, were treated as outcome variables. As trials with errors were excluded in order to be able to compute the network measures, associations between omission and commission errors with network measures were not examinable. Network measures, Age, and SART Task were treated as predictors in the models. Network measures were centered to reduce collinearity. Age and SART Task were coded in the same manner as described above. The network measures during the baseline were not examined as they were assumed to be task irrelevant. The network measures during the task and their interactions with Age and SART Task were specifically examined, where Age and SART Task were considered as control variables. Statistical results were summarized and reported in Table 2.

**TABLE 2:** Behavioral measures with network measures.

**Outcome variables**	**Predictors**	**β and CI**	**Significant interactions**	**β and CI**	**Significant pairwise comparison**
Mean RT	GE (theta)	β = −423.6, CI = [−899.4, 23.7]			
Mean RT	GE (alpha)	β = −315.1, CI = [−913.2, 297.8]			
Mean RT	GE (beta)	β = 496.8, CI = [−730.8, 1678.8]			
Mean RT	Mod (theta)	β = 745.0, CI = [−123.5, 1636.0]			
Mean RT	Mod (alpha)	β = 389.8, CI = [−433.5, 1191.9]			
Mean RT	Mod (beta)	β = 270.0, CI = [−1657.1, 2123.4]			
SDRT	GE (theta)	β = −280.9, CI = [−491.1, −63.8]^∗^			
SDRT	GE (alpha)	β = −96.4, CI = [−327.7, 143.9]			
SDRT	GE (beta)	β = 0.101.1, CI = [−436.7, 584.5]			
SDRT	Mod (theta)	β = 252.7 CI = [−66.0, 629.4]	Mod (theta) × Age × SART (Figure 1)	β = 403.0, CI = [151.6, 663.1]	Mod:Adol:Fixed (slope of Mod = 1206.1, CI = [610.4, 1801.9]^∗^) – Mod:Child:Fixed (slope of Mod = −405.1, CI = [−997.9, 187.9]), *p* = 0.001^∗^ Mod:Adol:Fixed – Mod:Adol:Random (slope of Mod = 104.4, CI = [−468.3, 677.0]), *p* = 0.004^∗^ Mod:Adol:Fixed – Mod:Child:Random (slope of Mod = 105.3, CI = [−621.9, 832.5]), *p* = 0.044^∗^
SDRT	Mod (alpha)	β = −166.4, CI = [−482.7, 158.4]			
SDRT	Mod (beta)	β = −799.8, CI = [−1567.5, 27.2]	Mod (beta) × Age (Figure 3)	β = 876.5, CI = [95.6, 1675.4]	Mod:Child (slope of Mod = −1676.3, CI = [−3026.7, −326.0]^∗^) – Mod:Adol (slope of Mod = 76.7, CI = [−793.3, 946.6]), *p* = 0.033^∗^
Mu	GE (theta)	β = 129.7, CI = [−698.3, 417.8]			
Mu	GE (alpha)	β = −133.9, CI = [−876.9, 578.4]			
Mu	GE (beta)	β = 307.6, CI = [−1121.0, 1707.6]			
Mu	Mod (theta)	β = 475.2, CI = [−579.2, 1602.1]			
Mu	Mod (alpha)	β = 528.5, CI = [−460.0, 1494.8]			
Mu	Mod (beta)	β = 869.4, CI = [−1416.1, 3234.9]			
Sigma	GE (theta)	β = −94.4, CI = [−336.0, 132.4]			
Sigma	GE (alpha)	β = −78.8, CI = [−353.1, 179.7]			
Sigma	GE (beta)	β = −247.1, CI = [−794.0, 306.9]			
Sigma	Mod (theta)	β = 18.6, CI = [−362.7, 447.2]	Mod (theta) × Age × SART	β = 510.6, CI = [163.8, 828.9]	Mod:Adol:Fixed (slope of Mod = 905.5, CI = [213.0, 1598.0]^∗^) – Mod:Child:Fixed (slope of Mod = −707.9, CI = [−1412.8, −3.01]^∗^), *p* = 0.009^∗^ Mod:Adol:Fixed – Mod:Adol:Random (slope of Mod = −276.0, CI = [−941.6, 389.7]), *p* = 0.020^∗^
Sigma	Mod (alpha)	β = −105.0, CI = [−481.9, 247.8]			
Sigma	Mod (beta)	β = −773.3, CI = [−1637.2, 122.1]			
Tau	GE (theta)	β = −310.9, CI = [−622.4, −23.2]^∗^			
Tau	GE (alpha)	β = −129.7, CI = [−512.1, 236.1]			
Tau	GE (beta)	β = 294.8, CI = [−513.1, 1002.5]			
Tau	Mod (theta)	β = 254.3, CI = [−315.4, 818.9]			
Tau	Mod (alpha)	β = −56.6, CI = [−555.9, 436.2]			
Tau	Mod (beta)	β = −374.4, CI = [−1544.2, 754.9]			
FFAUS	GE (theta)	β = −903.1, CI = [−1586.7, −211.8]^∗^			
FFAUS	GE (alpha)	β = 166.8, CI = [−599.7, 895.8]			
FFAUS	GE (beta)	β = 436.2, CI = [−1093.2, 1934.3]			
FFAUS	Mod (theta)	β = 1029.0, CI = [−83.5, 2151.7]			
FFAUS	Mod (alpha)	β = −510.4, CI = [−1486.7, 419.8]			
FFAUS	Mod (beta)	β = −523.0, CI = [−2894.4, 1883.2]			
SFAUS	GE (theta)	β = −4356.2, CI = [−7943.4, −473.3]^∗^			
SFAUS	GE (alpha)	β = −2072.9, CI = [−6556.4, 2361.4]			
SFAUS	GE (beta)	β = 5391.1, CI = [−3356.1 14423.1]			
SFAUS	Mod (theta)	β = 865.5, CI = [−5500.0, 7345.1]			
SFAUS	Mod (alpha)	β = −5613.3, CI = [−11091.6, 82.2]	Mod (alpha) × Age (Figure 2)	β = 6179.6, CI = [947.1, 11368.4]	Mod:Child (slope of Mod = −11792.9, CI = [−20490.4, −3095.4]^∗^) – Mod:Adol (slope of Mod = 566.3, CI = [−6193.9, 7326.6]), *p* = 0.028^∗^
SFAUS	Mod (beta)	β = −7653.7, CI = [−21731.2, 5397.7]	Mod (beta) × Age × SART (Figure 4)	β = 10723.1, CI = [956.7, 20353.7]	Mod:Child:Fixed (slope of Mod = −38294.6, CI = [−65478.8, 11110.3]^∗^) – Mod:Adol:Fixed (slope of Mod = 3355.9, CI = [−16420.5, 23132.4]^∗^), *p* = 0.034^∗^

**ADHD represents the ADHD Index and it is continuous data. For Age, −1 = children or Child, 1 = adolescents or Adol. SART represents the SART Task, and −1 = Random, 1 = Fixed. For Flanker, −1 = Incongruent or Inc., 1 = Congruent or Con. β represents the coefficient of a predictor variable. CI represents 95% Confidence Interval. ^∗^ p < 0.05 or CI does not contain 0. GE represents Global Efficiency, Mod represents Modularity, (theta) represents the theta band, (alpha) represents the alpha band, (beta) represents the beta band.**

#### Global Efficiency on Behavioral Measures

Global efficiency in the theta band was negatively associated with SDRT, tau, FFAUS, and SFAUS. Reduced RTV was associated with a greater increase in global efficiency in the theta band from the baseline to the task. No other significant associations or interactions were found between global efficiency in the three frequency bands and the behavioral measures.

#### Modularity on Behavioral Measures

##### Theta band

**FIGURE 1:**
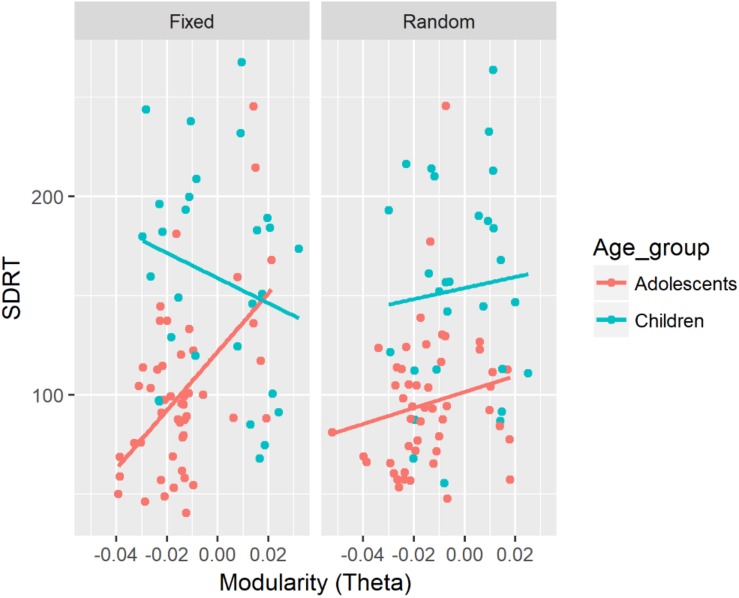
Associations between SDRT and modularity in the theta band for each age group and two versions of the SART. A similar association was also found for sigma.

There was a significant three-way interaction between modularity in the theta band, Age, and SART Task on SDRT (see Figure 1), and on sigma. In adolescents performing the fixed version of the SART, modularity in the theta band was positively associated with SDRT, and sigma. The strength of the negative associations in the fixed SART was greater for adolescents than for children in SDRT, and sigma. The strength of the negative associations in adolescents was also greater for the fixed than for the random version in SDRT, and sigma. Greater modularity in the theta band was associated with increased SDRT and sigma only for adolescents during the fixed SART.

There were no other significant interactions or main effects.

##### Alpha band

**FIGURE 2:**
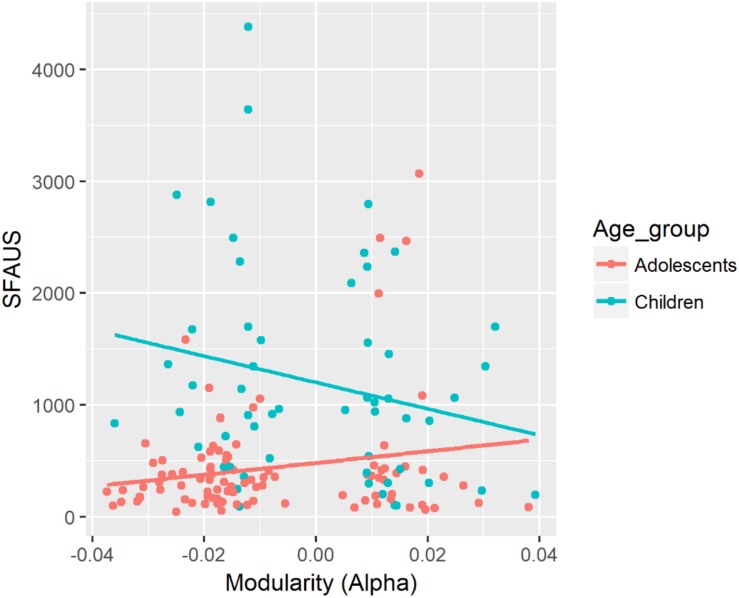
Associations between SFAUS from the FFT analysis and modularity in the alpha band for children and adolescents. In this figure, data from the fixed and random conditions of the SART have been aggregated for each age group.

There was a significant two-way interaction between modularity in the alpha band and Age on SFAUS (see Figure 2). There was a negative association between modularity in the alpha band and SFAUS for the child group, and the strength of this association was significantly greater for the child group than for the adolescent group. Children showed a greater decrease of SFAUS as an increase in modularity in the alpha band.

No other significant effects or interactions were found.

##### Beta band

**FIGURE 3:**
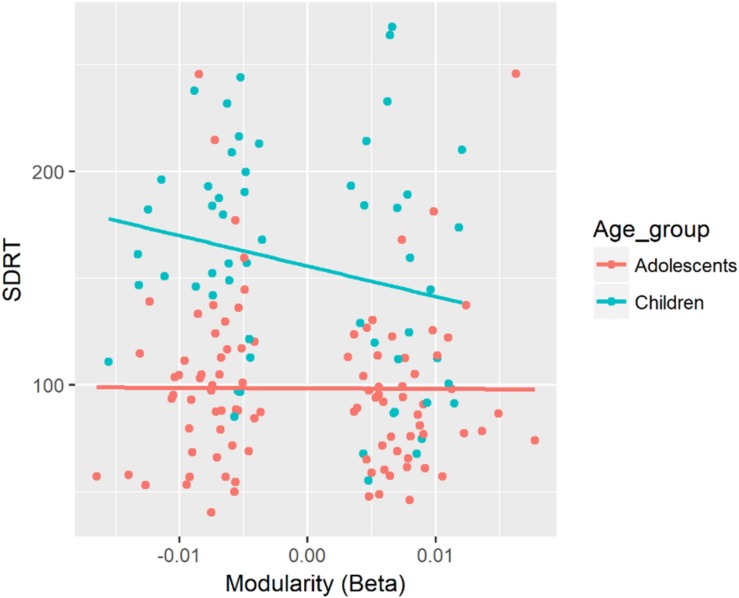
Associations between SDRT and modularity in the beta band for each age group. In this figure, data from the fixed and random conditions of the SART have been aggregated for each age group.

There was a significant two-way interaction between modularity in the beta band and Age for SDRT (see Figure 3). There was a negative association between modularity in the beta band and SDRT for the child group. The strength of this negative association was significantly greater for children compared with the adolescent group. Children showed a greater decrease of SDRT as an increase in modularity in the beta band.

**FIGURE 4:**
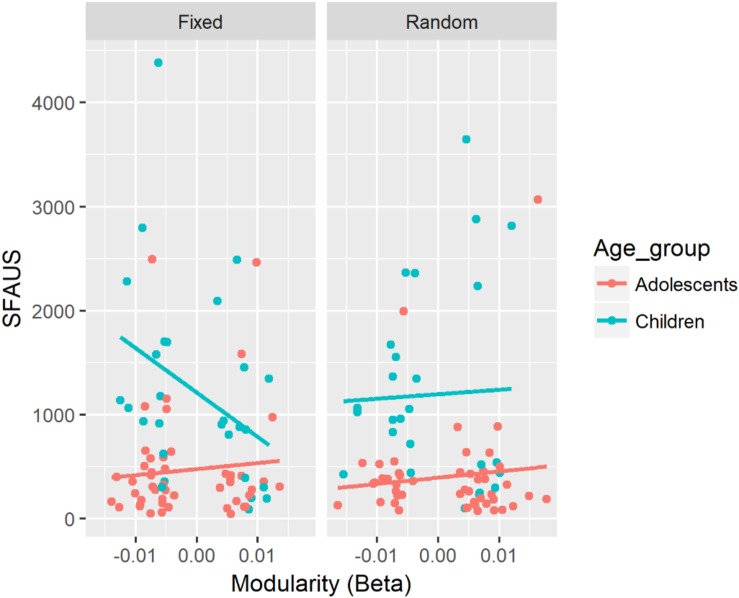
Associations between SFAUS and modularity in the beta band for each age group. The panel was split by the fixed and random versions of the SART.

There was a significant three-way interaction between modularity in the beta band, Age, and SART Task for SFAUS (see Figure 4). There was a negative association between modularity in the beta band and SFAUS for children on the Fixed SART. In children, the strength of this negative association was greater in the fixed version compared with the random version of SART. In the fixed version, the strength of this negative association was greater for children relative to adolescents. The other comparisons did not differ significantly. Children in the fixed version of SART showed a decrease in SFAUS as an increase in modularity in the beta band.

There were no other significant associations or interactions between modularity in the beta band and the behavioral measures.

### Network Measures – ADHD Index, Age, and SART Task

In the third type of model, network measures were inserted as outcome variables for the three frequency bands (theta, alpha, beta) and two time periods (baseline, task). Three predictors were inserted in the models: ADHD Index, Age, and SART Task. ADHD Index was centered to reduce collinearity. Age and SART Task were coded in the same manner as described above. Statistical results were summarized and reported in Table 3.

**TABLE 3:** Outcomes of analysis for network measures during the task period.

**Outcome variables**	**Predictors**	**β and CI**	**Significant interactions**	**β and CI**	**Significant pairwise comparison**
GE (theta)	ADHD	β = −0.0004, CI = [−0.0011, 0.0002]	ADHD × Age (Figure 5)	β = −0.0007, CI = [−0.0013, −0.0001]	ADHD:Adol (slope of ADHD = −0.0011, CI = [−0.0020, −0.0002]^∗^) − ADHD:Child (slope of ADHD = 0.0003, CI = [−0.0007, 0.0013]), *p* = 0.034^∗^
	Age	β = 0.0117, CI = [0.0024, 0.0211]^∗^			
	SART	β = −0.0013, CI = [−0.0043, 0.0017]			
GE (alpha)	ADHD	β = −0.0003, CI = [−0.0009, 0.0003]	Age x SART (Figure 6)	β = 0.0058, CI = [0.0020, 0.0096]	Adol:Fixed −Child:Fixed, *p* = 0.007^∗^
	Age	β = 0.0101, CI = [0.0020, 0.0187]^∗^			
	SART	β = −0.0010, CI = [−0.0048, 0.0027]			
GE (beta)	ADHD	β = 0.0000, CI = [−0.0001, 0.0002]			
	Age	β = −0.0011, CI = [−0.0036, 0.0016]			
	SART	β = 0.0005, CI = [−0.0016, 0.0026]			
Mod (theta)	ADHD	β = 0.0002, CI = [−0.0001, 0.0004]			
	Age	β = −0.0060, CI = [−0.0093, −0.0027]^∗^			
	SART	β = 0.0002, CI = [−0.0019, 0.0022]			
Mod (alpha)	ADHD	β = −0.0000, CI = [−0.0002, 0.0002]			
	Age	β = −0.0046, CI = [−0.0078, −0.0012]^∗^			
	SART	β = −0.0001, CI = [−0.0027, 0.0024]			
Mod (beta)	ADHD	β = −0.0001, CI = [−0.0003, −0.0000]^∗^			
	Age	β = −0.0002, CI = [−0.0018, 0.0015]			
	SART	β = −0.0005, CI = [−0.0016, 0.0005]			

**ADHD represents the ADHD Index and it is continuous data. For Age, −1 = children or Child, 1 = adolescents or Adol. SART represents the SART Task, and −1 = Random, 1 = Fixed. For Flanker, −1 = Incongruent or Inc., 1 = Congruent or Con. β represents the coefficient of a predictor variable. CI represents 95% Confidence Interval. ^∗^p < 0.05 or CI does not contain 0. GE represents Global Efficiency, Mod represents Modularity, (theta) represents the theta band, (alpha) represents the alpha band, (beta) represents the beta band.**

#### Main Effect of Age on Network Measures

Relative to children, adolescents performed the tasks with greater increases in global efficiency from the baseline to the task period in the theta and the alpha bands. Compared with children, adolescents showed a greater decline in modularity from the baseline to the task period in the theta, and alpha bands.

There were no other significant effects of Age.

#### Main Effect of ADHD Index on Network Measures

ADHD Index was negatively associated with modularity in the beta band during the task period.

There were no other significant effects of ADHD Index.

#### Interactions

**FIGURE 5:**
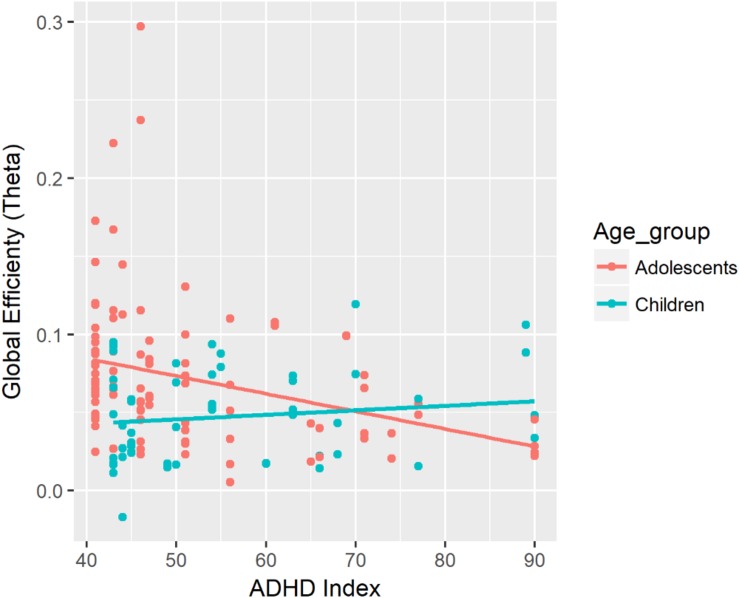
Associations between global efficiency in the theta band during the task and ADHD Index. In this figure, data from the fixed and random conditions of the SART have been aggregated for each age group.

There was a significant two-way interaction between ADHD Index and Age on global efficiency in the theta band during the task (see Figure 5). There was a negative association between ADHD Index and global efficiency in the theta band for adolescents. The strength of the negative association was greater for adolescents than for children. In adolescents, lower ADHD Index was associated with a greater increase in global efficiency from the baseline to the task in the theta band.

**FIGURE 6:**
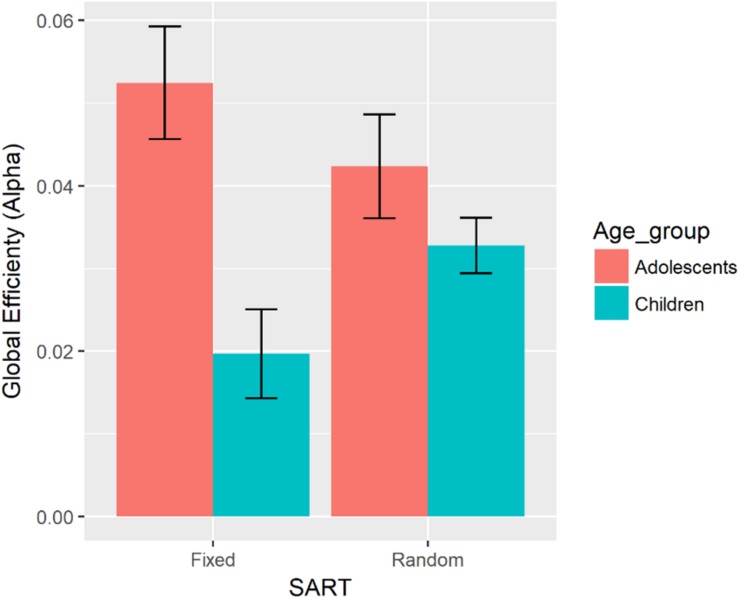
Bar graph representing global efficiency in the alpha band for each age group and the fixed and random versions of the SART. Error bars indicate the standard error.

There was a significant two-way interaction between Age, and SART Task on global efficiency in the alpha band during the task (see Figure 6). Compared with children, adolescents showed greater global efficiency in the alpha band during the task. There was no significant difference in global efficiency in the alpha band between adolescents and children in the random version of the SART.

There were no other significant interactions.

## Discussion

Children aged from 9 to 12 years and adolescents aged from 15 to 18 years completed the Fixed and Random SARTs while EEG was recorded. Higher ADHD Index was associated with greater RTV in all the RTV measures except for sigma. The same associations were found in both children and adolescents. As predicted, individuals with higher levels of ADHD symptoms tended to produce more variable responses. Adolescents showed a greater increase in global efficiency and decrease in modularity in the theta and alpha bands from the baseline to task periods, than the children. This suggests that adolescents may be better able to adjust their brain activity to perform the task smoothly relative to children, modifying brain activity be more integrated and less strongly segregated. A greater increase in global efficiency in the theta band was associated with all the RTV measures except for sigma. This indicates that having highly integrated brain activity is beneficial in producing more stable responses, possibly by combining information across the brain more efficiently. In adolescents only, a higher ADHD Index score was associated with a smaller increase from the baseline to the task period in global efficiency in the theta band. This may suggest that there might be a delay in maturation for adolescents with higher levels of ADHD symptoms. Their brains might be less efficient in shifting from the baseline to the task, which might lead to more variable responses.

### RTV and Global Efficiency

For stable performance, it may be important for the brain to be highly integrated. Global efficiency in the theta band during the task was significantly associated with SDRT, tau, FFAUS, and SFAUS, but it was not associated with mean RT, mu, or sigma. A greater increase of global efficiency in the theta band may be a key characteristic of the brain associated with RTV. The results suggest that greater integration of the brain during the task was associated with more stable performance. Greater integration of the brain, as measured by graph theoretical analysis, is thought to reflect efficiency of information processing (Micheloyannis et al., 2006; van den Heuvel et al., 2008; Bullmore and Sporns, 2012). The current results suggest that the more efficiently information is processed and integrated, the less variable responses become.

The theta band may play a crucial role in the brain’s ability to perform the task with stable responses, as global efficiency only in the theta band was associated with RTV measures. The importance of this theta band integration in cognitive task performance has been reported in studies applying the event-related networks approach to performance on the oddball task (Martin-Santiago et al., 2016) and the memory maintenance task (Toth et al., 2012). Sauseng et al. (2007) suggested that the theta band long range connectivity might play a role in integrating sensory information into executive control. The theta band long range connections were also suggested to coordinate information from various parts of the brain during the mental task to produce an output (Mizuhara et al., 2004; Berthouze et al., 2010). Therefore, greater integration of the brain in the theta band might be reflecting how efficiently the information across the brain is integrated before the output of responses. Among the RTV measures investigated in this study, only sigma was not significantly associated with global efficiency in the theta band. Considering that sigma is a measure of RTV without extremely slow responses (Luce, 1986), greater integration of the brain in the theta band during the task might be associated with those slow responses that are also measured through SDRT, tau, FFAUS and SFAUS. Reduced integration of the brain in the theta band might lead to more occurrences of those extremely slow responses as the information is not efficiently combined, resulting in a delayed response.

### Age Effects

From late childhood to late adolescence, the brain may be developing the ability to efficiently adjust functional connectivity for maximal task performance. Compared with children, adolescents showed a greater increase in global efficiency and decrease in modularity in the theta and alpha bands from baseline to performing the fixed and random SARTs. The results indicate that adolescents were adjusting their brains to be more integrated and strength of segregation to be decreased compared with children when performing the task. Bola and Sabel (2015) suggested that the network structure of the brain changes from the baseline to task active state by increasing the connectivity between modules, resulting in a more functionally integrated and less strongly segregated state during the cognitive process compared with baseline. This increase in integration and decrease in strength of segregation during the cognitive process has been found previously in adults (Toth et al., 2012; Cruzat et al., 2016; Martin-Santiago et al., 2016). Typically developing children showed greater global efficiency over the course of a naming task compared with children with reading difficulties, suggesting more efficient processing with greater integration (Vourkas et al., 2011). The current results of increased integration and decreased strength of segregation during the task in adolescents compared with the children might be linked with cognitive maturation. With cognitive maturation, individuals might be better able to smoothly reconfigure their brain connections to increase functional integration and decreased strength of segregation for more efficient cognitive processing.

Adolescents with higher levels of ADHD symptoms might have difficulty with the brain becoming more integrated when performing the task, which may possibly indicate a delay in maturation of the brain. Adolescents with lower levels of ADHD symptoms showed a greater increase of global efficiency in the theta band from the baseline to the task compared with adolescents with higher levels of ADHD symptoms. Previous studies have suggested that individuals with ADHD showed less brain integration, as measured by the characteristic path length and global efficiency (Ahmadlou et al., 2012; Lin P. et al., 2014; Liu et al., 2015). The association between ADHD Index and global efficiency in adolescents indicates that the level of functional integration in the theta band might explain the relationship between RTV and ADHD symptoms. Individuals with higher levels of ADHD symptoms might show lower levels of integration of the brain in the theta band during the task, which might lead to greater RTV especially with extremely slow responses. This association, however, was only found in adolescents, not in children. Considering that greater increase in global efficiency was observed in adolescents during the task compared with children, adolescents with higher levels of ADHD might show delays in maturation resulting in greater RTV and decreased global efficiency. This delay in maturation might become more apparent in adolescents, resulting in an association between ADHD Index and global efficiency in the theta band only in adolescents.

### RTV and ADHD Symptoms

As hypothesized, individuals with higher levels of ADHD symptoms showed more variable responses. The ADHD Index was positively associated with SDRT, tau, FFAUS, and SFAUS. The results were consistent with previous research and our prediction that higher levels of ADHD symptoms would be associated with greater RTV. In the Ex-Gaussian analysis, any extremely long responses, most likely representing lapses in attention during the task are captured by tau (Leth-Steensen et al., 2000). The strong association between tau and the ADHD Index suggests that those with elevated levels of ADHD symptoms might experience more lapses in attention during the cognitive task, resulting in increased RTV. This finding is similar to previous studies reporting that tau is a significant predictor of ADHD (Leth-Steensen et al., 2000; Buzy et al., 2009; Gmehlin et al., 2014; Karalunas et al., 2014; Lin H. Y. et al., 2014). Our results also showed that participants with elevated levels of ADHD symptoms performed the tasks with significantly greater FFAUS and SFAUS. FFAUS is thought to be a measure of sustained attention/cognitive control of attention, while SFAUS is thought to be a measure of arousal levels (Johnson et al., 2007, 2008). This greater RTV, indicated by both RTV frequency bands, is consistent with previous studies with participants with a formal clinical diagnosis of ADHD (Johnson et al., 2007, 2008; Karalunas et al., 2013; Adamo et al., 2014) and those with more severe symptoms of ADHD (Gomez-Guerrero et al., 2011; Mairena et al., 2012). As both FFAUS and SFAUS were associated with ADHD symptoms, individuals with more acute levels of ADHD might have difficulty in both sustaining attention and maintaining arousal levels.

### RTV and Modularity

The level of segregation might be related to RTV differently depending on the age of the participant. Only in the child group was modularity in the beta band negatively associated with SDRT. Additionally, a negative association between modularity in the beta band and SFAUS was found in children only in the fixed version of the SART. Children who showed less reduction of modularity during the task relative to baseline produced more stable responses. Previous research in adults has shown that less segregated brain activity is associated with better cognitive performance (de Haan et al., 2012; Douw et al., 2014). Our results, in contrast, suggested that having greater strength of segregated brain activity was associated with children producing more stable performance. There might be a benefit of having greater strength of segregated brain activity, which is only found during childhood. It has been assumed that the small-world network structure with a highly integrated and segregated network is the structure for the most efficient information processing (Tononi et al., 1998; Sporns et al., 2004). Stam et al. (2006) used graph theoretical analysis using EEG and examined Alzheimer’s disease. They showed that Alzheimer patients showed longer characteristic path length, suggesting the loss of small-world structure and implies the potential link with cognitive dysfunction. In children, higher modularity and greater segregation might be more beneficial in terms of efficient information processing. Alternatively, Kitzbichler et al. (2011) showed that modularity was reduced when the cognitive load was higher in the N-back task. It might indicate that those children with greater strength of segregated brain activity were the ones that felt the task to be easier. Further studies are needed to examine the dynamics of modular structures during cognitive tasks in children and adolescents, possibly using source localization techniques to capture more detailed network structures.

### Fixed and Random SART

An expectation for upcoming stimuli helped participants to inhibit their responses to No/Go stimuli, but it might not influence RTV. Participants made more commission errors in the random version of the SART compared to the fixed version, but no other behavioral measures differed between the two SART tasks. A previous study by O’Connell et al. (2009) compared the fixed and random version of the SART and showed no differences in SDRT, which was consistent with our study showing no difference in RTV, measured using SDRT, sigma, and tau, between the two versions of the task. Being able to predict the next stimulus did not help to produce stable responses observed.

Even though the participants showed similar levels of RTV between the fixed and random version of the SART, the brain responded differently to the two conditions. O’Connell et al. (2009) ERP study showed the slow potential in the fixed version, which is thought to reflect an expectation of the next stimuli. This study found that adolescents showed a greater increase in global efficiency in the alpha band during the task compared to children only in the fixed SART. The alpha oscillation has been linked to tonic or intrinsic arousal (Sadaghiani et al., 2010; Sadaghiani et al., 2012). The fixed SART is cognitively less demanding because the target digit is expected and this might lead to greater degrees of boredom compared with the random SART. Greater global efficiency in the alpha band in adolescents compared to children only in the fixed SART might suggest that adolescents are more capable of maintaining arousal levels in the less cognitively demanding task. In addition, this expectancy effect might explain modularity in the theta band being associated with reduced SDRT and sigma only in adolescents on the fixed version of SART. Adolescents might be able to decrease functional segregation of the brain in the fixed version of SART to produce less variable responses.

## Limitations

Participants in this study were not clinically diagnosed with ADHD, and the analysis was performed using the levels of ADHD symptoms as measured by the Conners 3 ADHD Index. The results could be different with a clinically diagnosed group. Another limitation of the study is the mixed use of the self-report and parent forms of Conners 3 across individuals. The study treated the ADHD Index from parents and self-report as the same variable, as they are highly correlated (Conners, 2008), however the findings related to the ADHD Index might vary if the same type of administration was used for all participants.

Our analysis was performed using surface level EEG recording with 64 electrodes. This allowed us to infer overall connectivity patterns of the surface of the whole brain, but with limited resolution in space. Further studies could consider using source localization methods, fMRI, or simultaneous EEG/fMRI recording to form more complex networks with a greater number of nodes. This would allow for investigations into small local networks existing within the large network and how the small networks communicate each other, such as in the study of hubs of networks (van den Heuvel and Sporns, 2013; Sporns and Betzel, 2016). This source level analysis might be able to identify more detailed patterns of brain connectivity associated with RTV and ADHD symptoms.

Another limitation of this study was the choice in defining the task period, which was 300–600 ms. It was assumed that this task period is associated with cognitive processes of humans, but this selection of the time window was set by choice rather than determined by a data driven approach. This process of deciding the time window might introduce subjective bias. Future studies could consider adopting different time windows or use a mass univariate approach (Groppe et al., 2011) to avoid defining a particular time window.

## Conclusion

This study found that a greater increase in global efficiency in the theta band from the baseline to task active status was negatively associated with RTV, where the more integrated the brain, the more stable the performance. We suggest that greater integration of the brain allows for smoother communication between different parts of the brain and subsequently more efficient information flow. As the level of integration was not associated with speed of responses, stability and speed of responses were influenced by different mechanisms in the brain. Adolescents showed greater increase in global efficiency and greater decrease in modularity in the theta and alpha bands during the task compared with children. As children develop into adults, their brains are more efficiently able to shift state as needed for the task at hand, possibly reflecting cognitive maturation. As predicted, individuals with higher levels of ADHD symptoms performed the SARTs with greater RTV. Through the measurement of different aspects of RTV, this research reveals that the general stability of responses is not simply linked to ADHD symptoms. Higher levels of ADHD symptoms might be associated with more lapses in attention (tau), greater difficulty in sustaining attention (FFAUS) and maintaining arousal levels (SFAUS), which may contribute to increased RTV. SDRT, tau, FFAUS, and SFAUS were associated with both ADHD symptoms and global efficiency in the theta band but not with sigma. With these similar patterns of associations, ADHD symptoms and global efficiency in the theta band might explain similar aspects of RTV. From the examination of associations between ADHD symptoms and network measures, only in the adolescent group was global efficiency in the theta band during the task associated with the ADHD Index. This suggests that adolescents with greater levels of ADHD might have a delay in maturation, thus exhibiting lower levels of integration activity and increased RTV.

## Data Availability Statement

The datasets generated for this study are available on request to the corresponding author.

## Ethics Statement

The University of Melbourne Human Research Ethics Committee, the Victorian Department of Education and Training, and the Catholic Education Office in the Archdiocese of Melbourne approved the study, in accordance with the 1964 Declaration of Helsinki. Parents and children provided written informed consent prior to each child’s participation in the study. Adult participants provided written informed consent prior to the study.

## Author Contributions

KM and KJ contributed to the design and implementation of the research, to the analysis of the results, and to the writing of the manuscript. MM helped with the analysis and the writing of the manuscript.

## Conflict of Interest

The authors declare that the research was conducted in the absence of any commercial or financial relationships that could be construed as a potential conflict of interest.
