# Supplementary material for: Electrophysiological Correlates of Response Time Variability During a Sustained Attention Task
Source: Front Hum Neurosci. 2019 Oct 15;13:363. doi: 10.3389/fnhum.2019.00363 (PMC6803451; doi:10.3389/fnhum.2019.00363)
Supplement: Supplementary file 1 [file Table_1.DOCX]

**Supplementary Material**

Table 4

*Analysis for network measures during the baseline period*

| Outcome Variables | Predictors | β and CI | Significant Interactions | β and CI | Significant pairwise comparison |
| --- | --- | --- | --- | --- | --- |
| GE (theta) | ADHD  Age  SART | β = -.0001, CI = [-.0002, .0001]  β = -.0034, CI = [-.0057, -.0016]*  β= .0010, CI = [-.0004, .0024] |  |  |  |
| GE (alpha) | ADHD  Age  SART | β = -.0002, CI = [-.0005, .0001]  β = -.0027, CI = [-.0068, -.0012]*  β= .0012, CI = [-.0007, .0031] |  |  |  |
| GE (beta) | ADHD  Age  SART | β = -.0001, CI = [-.0003, .0000]  β = -.0001, CI = [-.0021, .0021]  β= .0001, CI = [-.0013, .0010] |  |  |  |
| Mod (theta) | ADHD  Age  SART | β = .0000, CI = [-.0000, .0000]  β = -.0027, CI = [-.0035, -.0018]*  β= -.0003, CI = [-.0009, .004] |  |  |  |
| Mod (alpha) | ADHD  Age  SART | β = .0001, CI = [.0000, .0002]*  β = -.0022, CI = [-.0033, -.0010]*  β= .0001, CI = [-.0007, .0008] |  |  |  |
| Mod (beta) | ADHD  Age  SART | β = .0000, CI = [-.0000, .0001]  β = -.0015, CI = [-.0023, -.0007]*  β= .0001, CI = [-.0003, .0005] |  |  |  |

*Notes*. ADHD represents the ADHD Index and it is continuous data. For Age, -1 = children or Child, 1 = adolescents or Adol. SART represents the SART Task, and -1 = Random, 1 = Fixed. For Flanker, -1 = Incongruent or Inc, 1 = Congruent or Con. β represents the coefficient of a predictor variable. CI represents 95 % Confidence Interval. * *p* < .05 or CI does not contain 0. GE represents Global Efficiency, Mod represents Modularity, (theta) represents the theta band, (alpha) represents the alpha band, (beta) represents the beta band.
